# Usefulness of virtual scale endoscope for early gastrointestinal lesions

**DOI:** 10.1002/deo2.386

**Published:** 2024-06-18

**Authors:** Nobuhisa Minakata, Hiroaki Ikematsu, Fumiaki Kiyomi, Suma Inoue, Takashi Akutagawa, Takashi Watanabe, Tomonori Yano, Ryo Shimoda

**Affiliations:** ^1^ Department of Gastroenterology and Endoscopy National Cancer Center Hospital East Chiba Japan; ^2^ Department of Statistics and Data Center Clinical Research Support Center Kyushu Fukuoka Japan; ^3^ Department of Internal Medicine Division of Gastroenterology Saga University Saga Japan; ^4^ Department of Endoscopic Diagnostics and Therapeutics Saga University Hospital Saga Japan

**Keywords:** colonoscopy, endoscopic submucosal dissection, endoscopy, prospective study, size perception

## Abstract

**Objectives:**

For early gastrointestinal lesions, size is an important factor in the selection of treatment. Virtual scale endoscope (VSE) is a newly developed endoscope that can measure size more accurately than visual measurement. This study aimed to investigate whether VSE measurement is accurate for early gastrointestinal lesions of various sizes and morphologies.

**Methods:**

This study prospectively enrolled patients with early gastrointestinal lesions ≤20 mm in size visually. Lesion sizes were measured in the gastrointestinal tract visually, on endoscopic resection specimens with VSE, and finally on endoscopic resection specimens using a ruler. The primary endpoint was the normalized difference (ND) of VSE measurement. The secondary endpoints were the ND of visual measurement and the variation between NDs of VSE and visual measurements. ND was calculated as (100 × [measured size − true size] / true size) (%). True size was defined as size measured using a ruler.

**Results:**

This study included 60 lesions from April 2022 to December 2022, with 20 each in the esophagus, stomach, and colon. The lesion size was 14.0 ± 6.3 mm (mean ± standard deviation). Morphologies were protruded, slightly elevated, and flat or slightly depressed type in 8, 24, and 28 lesions, respectively. The primary endpoint was 0.3 ± 8.8%. In the secondary endpoints, the ND of visual measurement was −1.7 ± 29.3%, and the variability was significantly smaller in the ND of VSE measurement than in that of visual measurement (*p* < 0.001, F‐test).

**Conclusions:**

VSE measurement is accurate for early gastrointestinal lesions of various sizes and morphologies.

## INTRODUCTION

Advanced cancer in the gastrointestinal tract, such as the esophagus, stomach, and colon, is the leading cause of morbidity and mortality worldwide.^1^ Endoscopic resection (ER) has been widely performed for early‐stage gastrointestinal lesions to prevent death from advanced cancer in the gastrointestinal tract.^2^, ^3^, ^4^, ^5^ In determining ER methods (such as polypectomy, endoscopic mucosal resection, and endoscopic submucosal dissection) and surveillance intervals, lesion size is a crucial factor in early‐stage gastrointestinal lesions.^6^, ^7^, ^8^ Although biopsy forceps, hoods, or measuring forceps are sometimes used to measure lesion size accurately, this approach is time‐consuming and laborious; therefore, lesion size is measured visually.^9^, ^10^, ^11^ However, visual measurement can result in errors depending on conditions and bias in measurement values among endoscopists, leading to reproducibility and objectivity issues.^12^, ^13^, ^14^

Virtual scale endoscope (VSE) is a new gastrointestinal endoscope that displays virtual measures with a single push of a button.^15^ Previous reports have shown that VSE measurement provides more accurate measurements of size for silicone hemispherical polyps, ranging from 2.5 to 28.0 mm in diameter, than visual measurement.^16^ However, whether VSE measurement can accurately measure early‐stage gastrointestinal lesions of various morphologies and sizes in actual clinical settings remains unclear.

Therefore, this study aimed to determine whether VSE measurement provides accurate measurements for early gastrointestinal lesions of different morphologies and sizes.

## METHODS

### Study design

This multi‐center prospective observational study was conducted from April 2022 to December 2022. The Institutional Review Boards of all participating hospitals approved the study protocol.

### Inclusion and exclusion criteria

The inclusion criteria were as follows: patients with esophageal, gastric, or colorectal lesions resected endoscopically with a pretreatment diagnosis of ≤ 20 mm early‐stage lesion visually; those aged > 20 years; and those who provided written informed consent for participation in the study. Patients deemed to be ineligible for this study by their physicians were excluded.

### Procedures and measurements of lesion size

**FIGURE 1:**
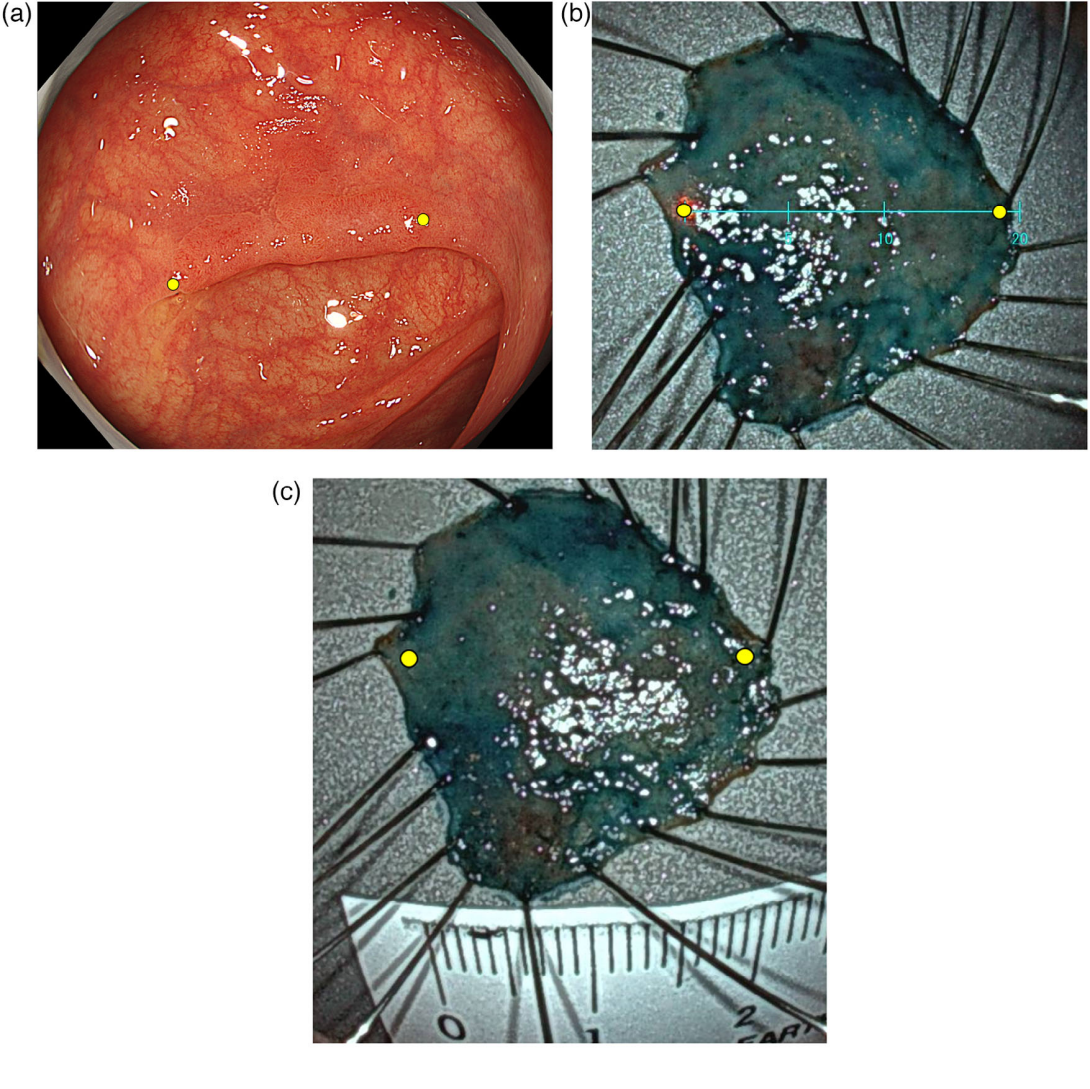
A representative case in measurements of lesion size. (a) The lesion size in the gastrointestinal tract measured visually during the treatment (length between two fixed points), (b) The lesion size on the endoscopic resection specimen measured using a virtual scale endoscope (length between two fixed points), and (c) The lesion size on the endoscopic resection specimen measured using a ruler (length between two fixed points).

All lesions were resected en bloc using endoscopic mucosal resection or endoscopic submucosal dissection. Six endoscopists, consisting of three experts and three non‐experts, participated in the study. Endoscopists who were accredited by the Japan Gastroenterological Endoscopy Society were defined as experts, and gastroenterologists who were not accredited by the Japans Gastroenterological Endoscopy Society were defined as non‐experts. Each endoscopist visually measured the lesion sizes between two fixed points in the gastrointestinal tract during the treatment. After the lesions were resected, they were stretched and fixed using needles; the lesion sizes were measured on the ER specimens using VSE and finally using a ruler. They were measured in 1‐mm increments (Figure 1). Pathological diagnosis was made according to World Health Organization criteria.^17^

### VSE and other setting

VSE is equipped with a red laser beam guide to calculate the distance between the endoscope tip and the lesion and a virtual scale of up to 20 mm is displayed on the monitor in real‐time. Adaptively changing the virtual scale length while varying the distance between the endoscope tip and the lesion was based on the triangulation method. The two types of scales to select from were linear and circular, with 5, 10, and 20 mm scales. Both scales were used to measure the size of all lesions. Lesions of > 20 mm in VSE measurement were estimated in size with reference to the virtual scale. The red laser and scale are shown or hidden by pressing a button on the endoscope.^15^

The virtual scale function operates using the combination of the dedicated endoscope EC‐760S‐A/M (Fujifilm) and the software for virtual scale EW10‐VM01 (Fujifilm). The endoscope is part of the ELUXEO system (Fujifilm), comprising the VP‐7000 processor and the BL‐7000 light source.^15^

### Outcomes

The primary endpoint was the normalized difference (ND) in the VSE measurement of the lesion on the ER specimen. In contrast, the secondary endpoints were the ND of the visual measurement of the lesion in the gastrointestinal tract and differences in the variability between NDs in the VSE and visual measurement. ND was defined as the relative accuracy of estimates of true lesion size and defined as follows: 100 × (measured lesion size − true lesion size) / true lesion size.^16^ True lesion size was defined as the size measured using a ruler on the ER specimen.

### Sample size calculation

The measurement precision (width of the confidence interval) of the mean for the primary endpoint was examined since this was not a confirmatory study.

The standard deviation (SD) of the ND for the VSE measurement was estimated to be 15, based on a previous study.^16^ With an SD of 15, 47 specimens were required to provide the width of the 95% confidence interval of the mean to be < 10, with at least 90% probability. In this study, 60 samples were required (20 each) since the measurements were obtained from three organs (esophagus, stomach, and colon).

### Statistical analysis

All variables are calculated as means and SDs or numbers and percentages. A 95% confidence interval of ND was constructed, and one sample t‐test or a signed rank test was used to evaluate if the mean of ND was 0 or the pseudo median of ND was 0, respectively. The Student's t‐test or one‐way analysis of variance (ANOVA) was used to compare the strata of factors. The variability of ND was compared between the NDs of the VSE and visual measurements using the F‐test. As an exploratory analysis, the primary endpoint was analyzed using the ANOVA with the endoscopist, organ, and morphology as fixed effects, and lesion size, which was measured using a ruler, as a covariate to evaluate the effect of the factors and covariate on the primary endpoint in a multivariate manner.

Statistical significance was set at *p* < 0.05. No correction was made for multiple comparisons because of the non‐confirmatory study design. All statistical analyses were performed using SAS version 9.4 (SAS Institute Inc.).

## RESULTS

This study included 60 lesions from patients at multi‐centers from April 2022 to December 2022. Twenty lesions each were present in the esophagus, stomach, and colon. The lesion size was 14.0 ± 6.3 mm (mean ± SD). Morphologies were protruded, slightly elevated, and flat or slightly depressed types in 8 (13.3%), 24 (40.0%), and 28 (46.7%) lesions, respectively. In total, 21 (35%) and 39 (65%) lesions were measured by experts and non‐experts, respectively (Table 1).

**TABLE 1:** Patient and lesion (*N* = 60) characteristics and endoscopist and institute details.

Patient characteristics	
Sex, male, *n* (%)	47 (78.3%)
Age (years), mean (SD)	71.7 (10.1)
Endoscopist and institute	
Endoscopist, expert, *n* (%)	21 (35.0%)
Institute, *n* (%)	
National Cancer Center East	33 (55.0%)
Saga University	27 (45.0%)
Lesion characteristics	
Organ, *n* (%)	
Esophagus	20 (33.3%)
Stomach	20 (33.3%)
Colon	20 (33.3%)
Size (mm)*, mean (SD)	14.0 (6.3)
Size by organ (mm), mean (SD)	
Esophagus	11.8 (5.2)
Stomach	17.6 (7.9)
Colon	12.8 (3.6)
Morphology, *n* (%)	
Protruded type	8 (13.3%)
Slightly elevated type	24 (40.0%)
Flat or slightly depressed type	28 (46.7%)
Morphology, detail, *n* (%)	
I	2 (3.3%)
Ip	2 (3.3%)
Is	2 (3.3%)
Isp	2 (3.3%)
IIa	22 (36.7%)
IIa+IIc	2 (3.3%)
IIb	3 (5.0%)
IIc	25 (41.7%)

*Size measured using a ruler.

Abbreviation: SD, standard deviation.

**FIGURE 2:**
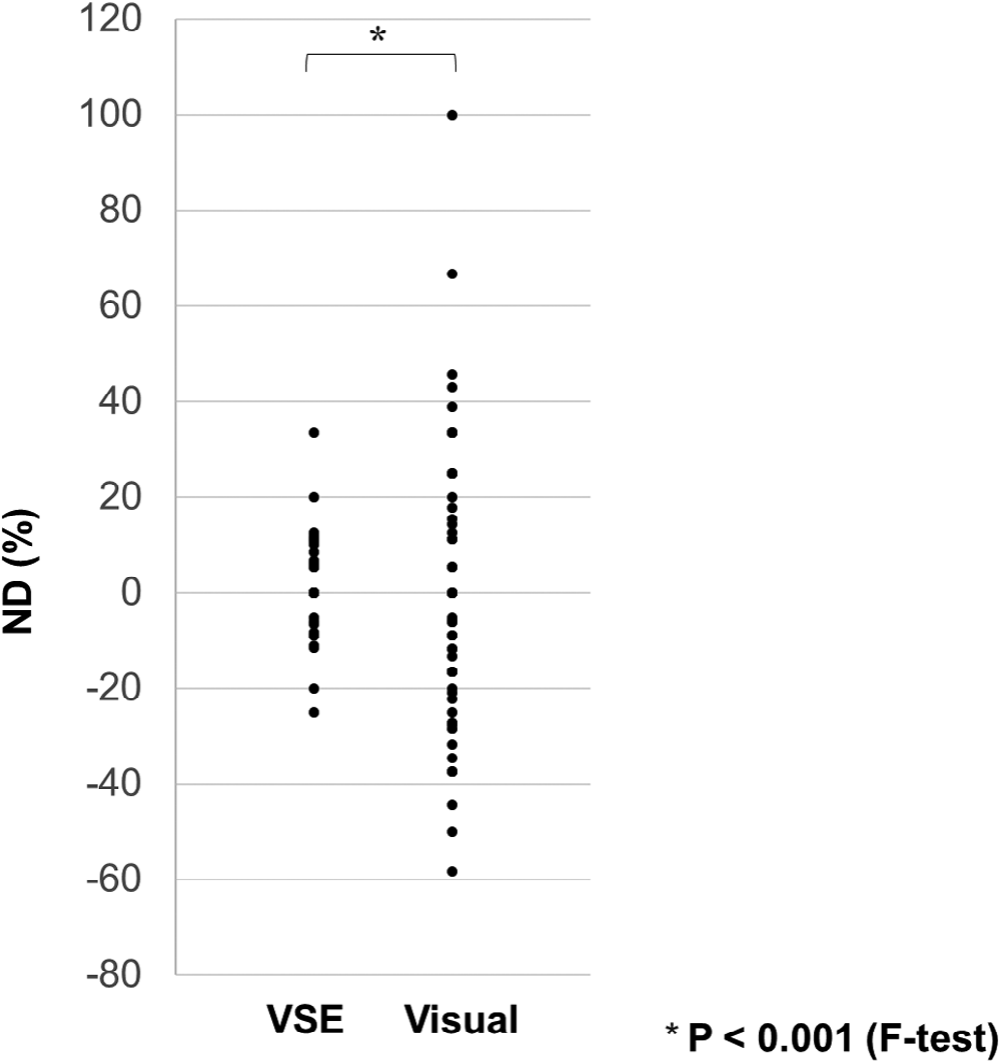
The variability was significantly smaller in the ND of the VSE measurement (SD = 8.8%) than in that of the visual measurement (SD = 29.3%; *p* < 0.001, F‐test). ND, normalized difference; VSE, virtual scale endoscope; SD, standard deviation.

In the primary endpoint, the ND of the VSE measurement was 0.3 ± 8.8% (Table 2), whereas that of the visual measurement in the secondary endpoints was −1.7 ± 29.3% (Table 3). The variability was significantly smaller in the ND of the VSE measurement (SD = 8.8%) than in that of the visual measurement (SD = 29.3%; *p* < 0.001, F‐test; Figure 2).

**TABLE 2:** The normalized difference of the virtual scale endoscope measurement (%).

	*N*	Mean (SD)	95% CI of the mean	*p*‐value^*^
All lesions	60	0.3 (8.8)	(‐1.9, 2.6)	0.766
Endoscopist				
Expert	21	3.3 (11.2)	(‐1.8, 8.4)	0.194
Non‐expert	39	‐1.2 (6.9)	(‐3.5, 1.0)	0.263
				*p* = 0.057^**^
Organ				
Esophagus	20	−1.3 (11.1)	(−6.5, 3.9)	0.607
Stomach	20	−0.0 (6.3)	(−2.9, 2.9)	0.999
Colon	20	2.3 (8.4)	(−1.6, 6.3)	0.234
				*p* = 0.428^**^
Morphology				
Protruded type	8	3.1 (7.7)	(−3.3, 9.6)	0.285
Slightly elevated type	24	0.8 (7.7)	(−2.4, 4.1)	0.600
Flat or slightly depressed type	28	−0.9 (10.0)	(−4.8, 3.0)	0.642
				*p* = 0.497^**^
Size				
<10 mm	15	0.6 (12.0)	(−6.0, 7.3)	0.837
10 ‐ 19 mm	36	1.4 (7.8)	(−1.3, 4.0)	0.306
≥20 mm	9	−4.2 (5.1)	(−8.1, ‐0.3)	0.125
				*p* = 0.236^**^

* One sample t‐test for the null hypothesis of “mean = 0” or signed rank test for the null hypothesis of “pseudo median = 0.”

** Student's t‐test or analysis of variance for comparing strata.

Abbreviations: CI, confidence interval; SD, standard deviation.

**TABLE 3:** The normalized difference of the visual measurement (%).

	*N*	Mean (SD)	95% CI of the mean	*p*‐value^*^
All lesions	60	−1.7 (29.3)	(−9.2, 5.9)	0.662
Endoscopist				
Expert	21	8.4 (26.7)	(−3.8, 20.5)	0.165
Non‐expert	39	−7.1 (29.6)	(−16.7, 2.5)	0.143
				p*p* = 0.050^**^
Organ				
Esophagus	20	−1.8 (33.4)	(−17.5, 13.8)	0.459
Stomach	20	−10.1 (26.8)	(−22.6, 2.5)	0.109
Colon	20	6.9 (26.2)	(−5.4, 19.2)	0.254
				p*p* = 0.189^**^
Morphology				
Protruded type	8	−11.9 (20.7)	(−29.2, 5.4)	0.147
Slightly elevated type	24	6.3 (35.2)	(−8.5, 21.2)	0.387
Flat or slightly depressed type	28	−5.6 (24.7)	(−15.2, 4.0)	0.240
				*p* = 0.197^**^
Size				
<10 mm	15	11.6 (39.0)	(−10.0, 33.2)	0.269
10 ‐ 19 mm	36	−3.8 (24.5)	(−12.1, 4.5)	0.356
≥20 mm	9	−15.2 (22.3)	(−32.3, 2.0)	0.086
				*p* = 0.073^**^

* One sample t‐test for the null hypothesis of “mean = 0” or signed rank test for the null hypothesis of “pseudo median = 0.”

** Student's t‐test or analysis of variance for comparing strata.

Abbreviations: CI, confidence interval; SD, standard deviation.

In the ND of the VSE measurement, experts tended to overestimate lesion size compared with non‐experts, although not significantly (*p* = 0.057, Student's t‐test). No significant differences were found in terms of organs (*p* = 0.428, one‐way ANOVA), morphologies (*p* = 0.497, Student's t‐test), and sizes (*p* = 0.236, one‐way ANOVA; Table 2). In the ND of the visual measurement, experts tended to overestimate lesion size, and non‐experts tended to underestimate lesion size, although not significantly (*p* = 0.050, Student's t‐test). No significant differences were observed in terms of organs (*p* = 0.189, one‐way ANOVA), morphologies (*p* = 0.197, Student's t‐test), and sizes (*p* = 0.073, one‐way ANOVA; Table 3). In the primary endpoint, ANOVA showed no significant differences between organs and morphologies. However, experts tended to overestimate lesion size more than non‐experts in the VSE measurement (*p* = 0.080, F‐test). No significant differences were found in the size measured using a ruler (*p* = 0.476, F‐test; Table 4).

**TABLE 4:** Analysis of variance (ANOVA) results of the normalized difference of the virtual scale endoscope measurement.

Fixed factor	Numerator d.f.	Denominator d.f.	*p*‐value^*^	
Endoscopist	1	53	0.080	
Organ	2	53	0.993	
Morphology	2	53	0.561	
Size measured using a ruler	1	53	0.467	

Estimated means				
Factor	Level	Estimated mean	95% CI	*p*‐value^**^
Endoscopist	Expert	3.5	(−0.7, 7.7)	0.102
	Non‐expert	−1.4	(−4.3, 1.6)	0.364
Organ	Esophagus	0.3	(−4.3, 4.9)	0.900
	Stomach	0.2	(−4.2, 4.5)	0.946
	Colon	0.6	(−4.6, 5.7)	0.821
Morphology	Protruded type	3.7	(−3.0,10.4)	0.269
	Slightly elevated type	0.2	(−3.9, 4.3)	0.937
	Flat or slightly depressed type	−0.5	(−4.5, 3.6)	0.813

* F‐test.

** t‐test for the null hypothesis of “mean = 0.”

Statistical methods: ANOVA, including endoscopist, organ, and morphology as fixed effects, and size, which was measured using a ruler as a covariate.

Abbreviations: CI, confidence interval; d.f., degree of freedom.

## DISCUSSION

This multi‐center prospective study using human specimens of various sizes and morphologies revealed three important findings. First, the mean ND of the VSE measurement was 0.3%, close to 0, indicating that it was accurate. Second, the mean visual measurement was −1.7% and was also close to 0, indicating that it was also accurate. Third, the variability was significantly smaller in the ND of the VSE measurement (SD = 8.8%) than in that of the visual measurement (SD = 29.3%) in the F‐test.

The mean ND of the VSE measurement was 0.3%, close to 0, indicating that it was accurate. Additionally, the SD of the ND of the VSE measurement was 8.8%, with slight variation. Previous reports on VSE, including one single‐center randomized control trial, have reported that target length on graph paper and pseudo‐polyps in a colorectal phantom could be measured with high accuracy, which aligns with our results.^15^, ^16^, ^18^ However, these reports were based on fixed size and morphologies targets, and the lack of various sizes and morphologies was an issue. This multi‐center study used human specimens of the most various morphologies and sizes ever reported. Furthermore, no previous reports have evaluated lesions not only in the colon but also in the esophagus and stomach. Therefore, these results are novel. Although VSE measurement on pseudo‐polyps in a colorectal phantom was also reported to underestimate the lesions by 12.5%, our results in this validation analysis show that the lesions can be measured accurately with minimal underestimation or overestimation.^16^ The measurement error in the VSE measurement becomes larger as the scope is displaced at an angle to the target.^15^ We consider the ease of facing and observing the lesion *ex vivo* as a factor that contributed to the accurate lesion measurements. Based on these results, the VSE measurement is expected to accurately measure lesion size regardless of size and morphology *in vivo*, as long as the lesion can be observed while facing it.

The mean visual measurement was −1.7%, which was close to 0, indicating that it was also accurate. However, the SD of the ND of the visual measurement was 29.3%, with a variation in each of them. No significantly different error factors in the visual measurement were observed in this exploratory analysis; however, certain lesions may not have been accurately measured. In fact, for the ND of the visual measurement, lesions of ≥20 mm tended to be underestimated, while lesions of <10 mm were usually overestimated. In contrast, the ND of the VSE measurements was accurate and showed slight variation at any size. Inaccurate lesion size measurements have been reported to be associated with female endoscopists, fewer colonoscopies performed in the past year, endoscopists with <10 years of practice, endoscopists with lower adenoma detection rates, and larger lesions.^9^, ^14^, ^19^, ^20^ Therefore, the VSE measurement may contribute to the improved accuracy in measuring lesion size under conditions that can lead to errors, such as those reported previously.

Expert endoscopists tended to slightly overestimate lesions. However, the difference was not significant, and when the lesion's actual size was considered, the difference was small and not significant in clinical practice. The lack of significant difference in size measured using a ruler in the ANOVA results suggests that no relationship exists between the size and the ND of the VSE measurement. Additionally, visual size measurement errors become larger as lesion size increases; however, our results suggest that VSE may be as accurate for large lesions as it is for small lesions with smaller measurement errors.^9^ Therefore, this is a novel finding.

This study has some limitations. First, the VSE measurements were made on resected specimens *ex vivo*. In the gastrointestinal tract, observation is challenging when facing lesions due to peristalsis and folds, and the VSE measurement is expected to be less accurate in vivo than ex vivo. Second, the conditions for measuring the lesion size differed between the VSE measurement ex vivo and the visual measurement in vivo. Finally, only cases with a maximum diameter of 20 mm in the preoperative visual examination were included because the virtual scale is limited to 20 mm, making it difficult to accurately measure lesions of > 20 mm. However, our results suggest that the VSE measurement can distinguish lesions of > 20 mm from those of ≤ 20 mm with reference to the virtual scale better than the visual measurement. Therefore, this ability may be useful in clinical practice if validated since the 20 mm cutoff is crucial in determining treatment strategies.

In conclusion, the VSE measurement is accurate for early gastrointestinal lesions of various sizes and morphologies ex vivo. Moreover, further study is needed to clarify whether the VSE measurement is also accurate for early gastrointestinal lesions in vivo.

## CONFLICT OF INTEREST STATEMENT

Hiroaki Ikematsu is an Associate Editor of *DEN Open*. The other authors declare no conflict of interest.
